# Targeting growth hormone function: strategies and therapeutic applications

**DOI:** 10.1038/s41392-019-0036-y

**Published:** 2019-02-08

**Authors:** Man Lu, Jack U. Flanagan, Ries J. Langley, Michael P. Hay, Jo K. Perry

**Affiliations:** 1 Liggins Institute, University of Auckland, Auckland, New Zealand; 2 Auckland Cancer Society Research Centre, School of Medical Sciences, University of Auckland, Auckland, New Zealand; 3 Maurice Wilkins Centre for Molecular Biodiscovery, Auckland, New Zealand; 4 Department of Molecular Medicine and Pathology, School of Medical Sciences, University of Auckland, Auckland, New Zealand

**Keywords:** Endocrine system and metabolic diseases, Molecular medicine, Drug development, Drug screening

## Abstract

Human growth hormone (GH) is a classical pituitary endocrine hormone that is essential for normal postnatal growth and has pleiotropic effects across multiple physiological systems. GH is also expressed in extrapituitary tissues and has localized autocrine/paracrine effects at these sites. In adults, hypersecretion of GH causes acromegaly, and strategies that block the release of GH or that inhibit GH receptor (GHR) activation are the primary forms of medical therapy for this disease. Overproduction of GH has also been linked to cancer and the microvascular complications that are associated with diabetes. However, studies to investigate the therapeutic potential of GHR antagonism in these diseases have been limited, most likely due to difficulty in accessing therapeutic tools to study the pharmacology of the receptor in vivo. This review will discuss current and emerging strategies for antagonizing GH function and the potential disease indications.

## Growth hormone overproduction: the current state of suppressive treatments

Emerging therapies are offering an expanded toolkit for combatting the effects of human growth hormone overproduction. Human growth hormone (GH) is a major driver of postnatal growth; however, systemic or localized overproduction is implicated in the aberrant growth disease acromegaly, cancer, and diabetes. In this review, researchers led by Jo Perry, from the University of Auckland, New Zealand, discuss strategies that either inhibit GH production, block its systemic receptor, or interrupt its downstream signaling pathways. The only licensed GH receptor blocker is pegvisomant, but therapies are in development that include long-acting protein and antibody-based blockers, and nucleotide complexes that degrade GHR production have also shown promise. Studies investigating GHR antagonism are limited, partly due to difficulty in accessing therapeutic tools which block GHR function, but overcoming these obstacles may yield advances in alleviating chronic disease.

## Introduction

Human growth hormone (GH) is a peptide hormone that is secreted from the anterior pituitary. It has a central function of regulating postnatal growth and metabolism and exhibits pleiotropic effects on various human tissues. Chronic hypersecretion of GH into the circulation, usually from a GH-secreting pituitary adenoma, is classically associated with acromegaly, a debilitating disease characterized by excessive skeletal growth, soft tissue enlargement, insulin resistance, and cardiovascular and gastrointestinal morbidities.^1^ Increased GH levels have also been implicated in cancer and diabetes.^2–5^ Pegvisomant, a GH analog, is the only clinically used antagonist of the GH receptor (GHR).^6,7^ However, other antagonists are in clinical trials or preclinical development. This review will focus on current strategies for antagonizing GH function and the related disease indications and will discuss considerations associated with an increasingly complex GH signal transduction network. Due to space limitations, reviews have been used in the place of original articles in some instances.

## GH secretion and physiological function

**Fig. 1:**
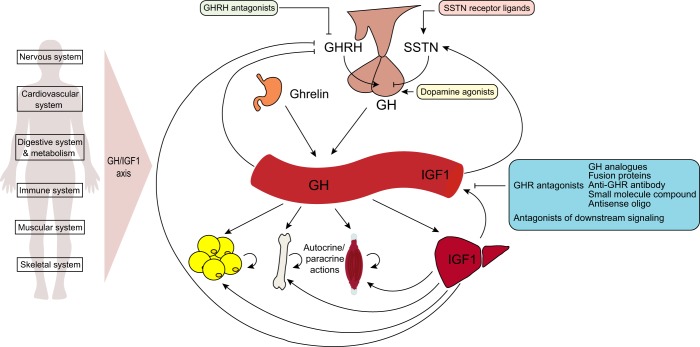
Endocrine regulation of GH and therapeutic blockade. GH is secreted from the anterior pituitary under the control of hypothalamic hormones, growth hormone releasing hormone (GHRH) and somatostatin (SSTN), and ghrelin, which is predominantly secreted in the stomach. Endocrine secretion of GH impacts numerous physiological systems with wide-ranging effects in various tissues. GH is also expressed in extrapituitary tissues in which it has localized autocrine/paracrine effects. Strategies to antagonize GH signaling are shown and are described in detail below

GH is released from the somatotroph cells of the anterior pituitary in a pulsatile fashion. Release is primarily regulated by the hypothalamic hormones, growth hormone-releasing hormone (GHRH; positive regulation), and somatostatin (negative regulation) (Fig. 1).^8^ GHRH is a peptide hormone that interacts with a G protein-coupled receptor (GHRHR) in somatotroph cells to activate the cAMP signaling pathway, which leads to increased *GH* mRNA transcription and release. GHRH upregulates the pituitary-specific POU homeodomain transcription factor, Pit-1, which in turn, transcriptionally upregulates the *GH1*, *GHRHR* and *Pit-1* genes (auto-upregulation). Activation of GHRHR signaling in somatotroph cells also induces the release of GH from secretory vesicles as a result of the influx of extracellular Ca^2+^.^8^ A complex series of short and long feedback loops negatively regulates GH secretion. Increased levels of GH and IGF1 in the circulation stimulate the release of somatostatin, which interacts with somatostatin receptors and negatively regulates GH secretion from the anterior pituitary.

GH secretion is also influenced by ghrelin, a GH secretagogue that is produced primarily by the endocrine cells of the stomach, but also by the intestinal tract and hypothalamus.^9^ In addition, secretion is regulated by thyroid hormones, leptin, androgens, and estrogen. Other key stimuli for secretion include nutrition, exercise, body composition, and the onset of deep sleep.^10–13^ Distinct sex-specific secretion patterns are apparent.^14,15^

Once released into the circulation, GH binds and activates the cell-surface GHR, as well as the related prolactin receptor in target tissues such as liver, muscle, bone, and adipose tissue (Fig. 1). It is the key regulator of insulin-like growth factor 1 (IGF1), which is secreted from target tissues, particularly the liver. Increased serum GH and IGF1 produce feedback loops that lead to inhibition of GHRH, release of somatostatin, and consequently inhibition of GH secretion from the pituitary. Whereas the endocrine system is the main secretory pathway, GH is also expressed in many extrapituitary tissues in which it has autocrine and paracrine effects.^4,16,17^

The primary function of GH is to promote postnatal longitudinal growth. It induces bone growth and is involved in the regulation of lipid, carbohydrate, nitrogen, and mineral metabolism and electrolyte balance. It increases lipolysis in adipocytes and decreases body fat; it increases amino acid uptake and nitrogen retention in muscle and maintains muscle mass and strength.^8,18^ GH has effects on the immune system, cardiovascular system, neurogenesis and the central nervous system, and aging.^3,19–21^ As a consequence, abnormal GH secretion has the potential to impact multiple tissues and organs. In particular, GH hypersecretion leads to gigantism in childhood and acromegaly in adults, whereas congenital disruption of GH signaling causes short stature and in rare cases Laron syndrome. In adults, deficiency is known as GH deficiency syndrome.

## Growth hormone receptor signal transduction

**Fig. 2:**
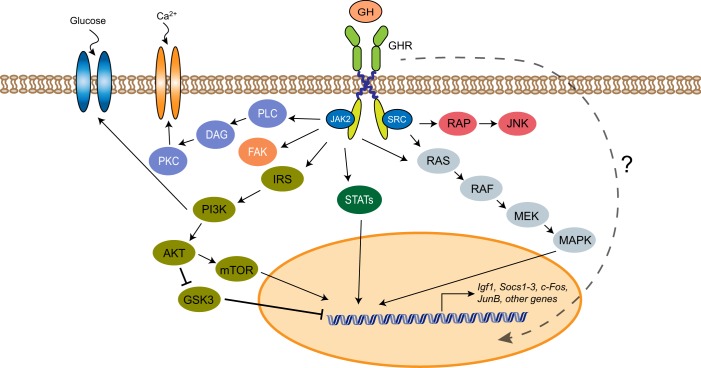
GHR signal transduction. A predimerized GHR interacts with the GH ligand and activates the associated kinases, JAK2 and SRC. Key signal transduction pathways activated by the GHR include the JAK-STAT, MEK/MAPK, PI3K/AKT/mTOR, and PLC/DAG/PKC pathways. The GHR can also translocate to the nucleus (dotted line), but the function remains unclear. GHR growth hormone receptor, GH growth hormone, JAK2 janus kinase 2, SRC SRC proto-oncogene, STAT signal transducer and activator of transcription, MEK mitogen-activated protein kinase kinase, MAPK mitogen-activated protein kinase, PI3K/AKT/mTOR phosphatidylinositol 3-kinase/AKT/mammalian target of rapamycin, GSK3 glycogen synthase kinase 3, IRS insulin receptor substrate, PLC/DAG/PKC phospholipase C/diacylglycerol/protein kinase C, FAK focal adhesion kinase, RAP rap guanine nucleotide exchanger

The GHR is a type I cytokine receptor that lacks intrinsic kinase activity and requires recruitment of the nonreceptor tyrosine kinase, Janus kinase 2 (JAK2), for activation.^2,3,22,23^ Substantial evidence also supports the concept that SRC family kinases, in particular LYN, are recruited to the receptor. These kinases participate in GHR signal transduction.^2,3^ A predimerized GHR homodimer interacts with the GH ligand through two binding sites, which have different affinities for the receptor. Binding leads to a rotational change in the receptor transmembrane domain, which leads to transphosphorylation and activation of two JAK2 molecules that are associated with the cytoplasmic domain of the receptor.^24,25^ Phosphorylated JAK2 then phosphorylates tyrosines in the cytoplasmic domain of GHR, and this facilitates recruitment of signaling molecules to the receptor. The primary signaling pathway activated by GH is the JAK-STAT (signal transducer and activator of transcription) pathway (Fig. 2). The STAT molecules that are activated by GH signaling are STAT1, 3, 5a, and 5b. Other key signaling pathways that are utilized are the mitogen-activated protein kinase (MAPK) and phosphatidylinositol 3-kinase/AKT/mammalian target of rapamycin (PI3K/AKT/mTOR) pathways, as well as SH2B1β, a scaffold protein that interacts with JAK2 and mediates GH-induced changes in the cytoskeleton.^22^ The GHR has also been observed to rapidly translocate to the nucleus following activation, but its role there remains unclear.

Once activated, GHR signal transduction is downregulated by suppressor of cytokine signaling proteins 1–3 (SOCS1–3); these are negative regulators that facilitate the ubiquitination and degradation of the receptor. Downregulation of the receptor also occurs through dephosphorylation by several protein tyrosine phosphatases and protein inhibitor of activated STATs (PIAS).^2^

Activation of the GHR and GHR-stimulated signal transduction pathways has been comprehensively reviewed elsewhere, and we refer the reader to recent reviews for a more detailed description of GHR-mediated signaling.^2,3,18,22,26^

**Fig. 3:**
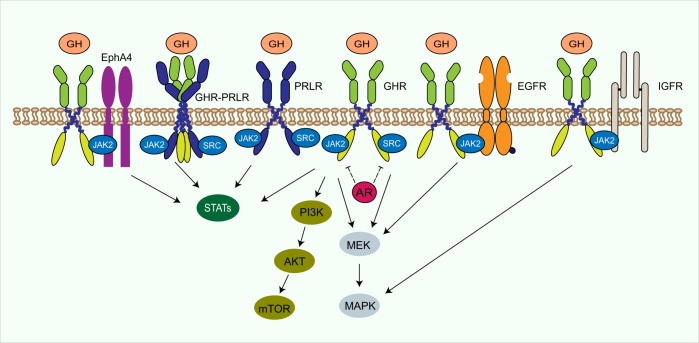
GHR crosstalk. In addition to the GHR, GH can bind and activate the PRLR, and the GHR can form heteromultimers with PRLR. Furthermore, GHR cross-talks and/or forms complexes with several other growth factor and hormone receptors, such as EphA4, EGFR, and IGF1R, which enhances the stimulation of downstream signaling pathways. GHR growth hormone receptor, GH growth hormone, JAK2 janus kinase 2, STAT signal transducer and activator of transcription, MEK mitogen-activated protein kinase kinase, MAPK mitogen-activated protein kinase, PI3K/AKT/mTOR phosphatidylinositol 3-kinase/AKT/mammalian target of rapamycin, PRLR prolactin receptor, EGFR epidermal growth factor receptor, IGF1R insulin-like growth factor 1 receptor, AR androgen receptor

In addition to activating the GHR, human GH can bind and activate a second cytokine receptor, the prolactin receptor.^27^ The GHR also cross-talks and/or forms complexes with several other growth factor and hormone receptors (Fig. 3). GH increases the phosphorylation of the epidermal growth factor receptor (EGFR) and promotes downstream ERK signaling.^28–30^ It has been proposed that a GHR-JAK2-IGF1 receptor complex is formed, which is activated by GH stimulation and inhibited by a soluble IGF1 receptor extracellular domain fragment.^31,32^ The androgen receptor also cross-talks with GH signaling at the level of STAT5 and SOCS2 in prostate cancer cells.^33^ More recently it has been demonstrated that EphA4, a member of the Eph family of receptor tyrosine kinases, may interact with the GHR.^34^ EphA4 knockout mice have dramatically reduced body size and impaired GHR signaling: they have normal levels of *GH* mRNA expression in the pituitary but have reduced plasma IGF1 and reduced *IGF1* mRNA expression in the liver. The authors demonstrated that EphA4 forms a complex with GHR and JAK2 and enhances IGF1 production in response to GH.^34^ Furthermore, recent studies from Stuart Frank’s laboratory have shown that the GHR and prolactin receptor form complex heteromultimeric structures that may affect GH signal transduction in some cell lines.^35,36^

## GH excess: disease indications

### Acromegaly

One of the most well-described diseases associated with excess GH is acromegaly, a chronic disease that is generally caused by benign pituitary adenomas, with rare exceptions that include secretion from tumors at other sites.^37^ Patients may exhibit clinical features, such as abnormal growth of the hands, feet and facial features, and enlarged organs. Other pathologic features linked to effects of excessive GH may be observed, such as vertebral deformities and abnormal calcium levels, increased risk of cardiovascular disease, respiratory comorbidities, and glucose intolerance.^1,38,39^ Hypersecretion of GH leads to elevated concentrations of circulating IGF1 in acromegalic patients, and these two hormones may have different metabolic features and target tissue responses.^40^

Treatment options for acromegaly include surgery, medical therapy and radiotherapy. The aim of treatment is to reduce both GH and IGF-1 to within normal limits.^37,40,41^ The primary choice for therapy is surgical removal of the tumor; however, there are some situations in which this may not achieve an optimal outcome, for example in the case of larger tumors with significantly high levels of GH. Therefore, more than one intervention may be necessary to achieve symptom remission and GH/IGF1 normalization. Medical therapies are an important treatment option, particularly for patients who may not be suitable for surgery or those with persistently high levels of GH/IGF1 following surgery. There are three main types of medical therapy, including somatostatin receptor ligands (SRL), GHR antagonists and dopamine agonizts^42,43^ (Table 1). The GHR antagonist pegvisomant is an analog of GH that competes with GH for receptor binding and consequently blocks GHR signal transduction. SRLs and pegvisomant are described in more detail in the following sections. Other strategies include dopamine agonists that bind to the dopamine receptors in the pituitary gland and block the secretion of prolactin and GH. These agents may benefit patients in whom hypersecretion of both prolactin and GH occurs. Radiotherapy is another therapeutic option but is reserved for aggressive tumors that are not controlled by surgery or medical treatment due to the high risk of hypopituitarism and other complications.^37,41^

**Table 1:** GH signaling inhibitors

Strategy/drug name	Target	Company
*SSTN receptor ligands*		
Octreotide LAR^42,43^	GH secretion	Novartis; clinical use
Lanreotide autogel^42,43^	GH secretion	Ipsen; clinical use
Pasireotide LAR^42,43^	GH secretion	Novartis; clinical use
Octreolin^42,43^	GH secretion	Chiasma; clinical development
Intravail Octreotide ProTek^42,43^	GH secretion	Aegis; clinical use
Somatoprim (DG3173)^42,43^	GH secretion	Aspireo; clinical development
*Dopamine agonist*		
Cabergoline^42,43^	PRL/GH secretion	Par Pharmaceutical; clinical use
GHRH antagonists^111^	GH secretion	Preclinical development
*GH analogs*		
Pegvisomant/B2036^6,114^	GHR	Pfizer; clinical use
GH-G120R^118^	GHR/PRLR	
*Fusion proteins*		
Antagonist–GHBP fusion^128^	GHR	Asterion Ltd.; clinical development
*Antisense oligonucleotide*		
ATL1103 (Atesidorsen)^132^	GHR	Antisense Therapeutics Ltd.; clinical development
*Anti-GHR antibody*		
Anti-GHRcyt-mAb^133,137^	GHR	
GF185^136^	GHR	
RN172^135^	GHR	Pfizer; preclinical
CG-86^134^	Ghr (porcine)	
*Small molecule compound*		
BVT-A^138^	Unknown	
*GH signaling pathway inhibitors*		
JAK-STAT, MAPK, PI3K/AKT/mTOR^140–144^		

*GHR* growth hormone receptor, *SSTN* somatostatin, *PRLR* prolactin receptor, *PRL* prolactin, *JAK-2* Janus kinase 2, *STAT* signal transducer and activator of transcription, *MAPK* mitogen-activated protein kinase, *PI3K/AKT/mTOR* phosphatidylinositol 3-kinase/AKT/mammalian target of rapamycin

### Cancer

There is a growing body of evidence that implicates GH in multiple cancer types, particularly breast, colon and endometrial cancer. Individuals with GH resistance, as seen in Laron syndrome, a rare genetic condition resulting from an inactivating mutation in the GHR, are protected from cancer and diabetes.^44–46^ Conversely, patients with acromegaly have a higher risk of developing certain cancers, although the extent of that risk remains a topic of debate with conflicting observations.^47,48^ This is partly due to limitations in the ability to quantify the risk of cancer in patients with a rare disease and differences in the methodological approaches. Recent nationwide cohort studies in Italy and Denmark both found an increased cancer risk in patients with acromegaly.^48,49^ This was supported by a meta-analysis of 23 studies, which observed a slightly elevated overall risk of cancer in these patients.^49^

Such observations are supported by numerous in vitro and in vivo studies that have demonstrated multiple effects on tumor development.^4,47,50,51^ GH accelerates tumor progression through autocrine/paracrine effects on cancer cell behavior and neighboring cells within the tumor microenvironment. These effects include promoting cell survival/proliferation,^52,53^ migration/invasion,^53,54^ oncogenic transformation,^55,56^ and epithelial-to-mesenchymal transition,^54,57,58^ as well as promoting tumor angiogenesis^59^ and lymphangiogenesis^60^ and enhancing a cancer stem cell-like phenotype.^57,61^ These effects have been observed in multiple tumor types, including breast, endometrial, and hepatocellular carcinomas, and lung cancer, melanoma, prostate cancer, and colon cancer.^17,47,57,61–63^ The effects are mediated through altered transcription of numerous genes that are associated with various aspects of cancer progression. A recent study demonstrated that disrupted GH signaling is associated with elevated p53 in colon tissue in humans and mice and that GH may act as a tumor promoter by suppressing p53, PTEN, and APC levels.^64^ In addition, GH regulates the function of other receptors that promote cancer progression.^65,66^

Single-nucleotide polymorphisms in GH-related genes are associated with the risk of developing osteosarcoma, breast, and colon cancer.^67–69^ Recently, a P495T variant of the GHR that is associated with increased incidence of lung cancer in various ethnic groups was shown to prolong GH signaling, which leads to increased expression of genes associated with tumor proliferation and epithelial-to-mesenchymal transition.^70^ The amino acid change at position 495 impairs SOCS2 binding and leads to a reduced downregulation of the receptor.

Such studies support the rationale for testing GHR antagonism in cancer, but only a small number of preclinical studies have used pegvisomant in an oncology setting. Single agent growth-inhibitory effects have been reported in breast, colon, and meningioma tumor xenografts, which suggests that GHR antagonism as a monotherapy may have efficacy in some tumor types.^71–74^ However, it is clear from these studies that not all tumor types tested were responsive, and currently the field is lacking diagnostic biomarkers capable of predicting response.

As with many targeted therapies, improved anticancer responses may be observed with combination therapeutic approaches. In this regard, GH promotes chemo- and radioresistance, and GHR antagonism/suppression may be beneficial when combined with radiotherapy and certain chemotherapeutic drugs.^47,75–77^ Recently, we reported that GH promotes radioresistance in cancer cell lines^78^ and that pegvisomant suppresses endometrial tumor regrowth following radiotherapy in a xenograft model, which highlights the potential utility of this combinational approach and identifies GHR antagonism as a potential molecularly targeted radiosensitizing strategy.^76^ This finding is of interest because for many common cancers, adding molecularly targeted agents to radiotherapy can increase preclinical cure rates,^79–81^ and currently the only targeted agent clinically approved for this application is the EGFR antagonist, cetuximab.

### Diabetes mellitus

Diabetes mellitus is a chronic metabolic disorder that is characterized by elevated blood glucose caused by deficiency or resistance to insulin. Glucose homeostasis is primarily regulated by insulin, which lowers blood glucose by increasing the uptake into cells and increasing its utilization and storage as fat and glycogen in peripheral tissues. Insulin also decreases gluconeogenesis (synthesis of glucose from noncarbohydrate carbon substrates) and glycogenolysis (breakdown of glycogen). GH has well-described diabetogenic actions. In particular, GH opposes the actions of insulin: it increases glucose production through gluconeogenesis and glycogenolysis in the liver and kidney, suppresses glucose uptake in adipose tissues, and is lipolytic. In healthy adults and adolescents during puberty, increased GH levels impair glucose tolerance and induce insulin resistance.^82,83^ Conversely, adults with Laron syndrome have reduced circulating IGF1 and increased insulin sensitivity.^45,46,84^ This is supported by studies in mice^85^ and dogs.^86^ Blocking the effects of GH in patients with acromegaly improves diabetes and glucose metabolism. However, a one-month treatment with a GHR antagonist had no effect on insulin sensitivity in insulin-resistant nondiabetic men.^87^

In addition to systemic effects on glucose metabolism and insulin sensitivity, GH may contribute to diabetes-associated complications, such as diabetic retinopathy and diabetic renal disease, through localized expression and autocrine/paracrine effects in tissues,^88–93^ although published research in these areas is limited. Diabetic retinopathy is one of the most frequent complications of diabetes and is a leading cause of blindness. The proliferative form is a more advanced stage of the disease and is characterized by retinal neovascularization. Extrapituitary expression of GH has been detected in the human retina and vitreous fluid,^94^ and GH has been demonstrated to directly stimulate the proliferation of human retinal microvascular endothelial cells in vitro.^95^ In vivo, ischemia-induced retinal neovascularization was inhibited in transgenic mice expressing a GH antagonist gene.^96^ Increased serum levels of GH and IGF1 have been observed in type 2 diabetes mellitus (T2D) patients with proliferative diabetic retinopathy compared to T2D patients with nonproliferative retinopathy or with no evidence of this complication.^97^

An increased prevalence of proliferative retinopathy is also observed in patients with acromegaly.^98^ Whether GHR-targeted strategies will be an effective treatment for retinopathy is still a matter of debate: a small clinical study using the GHR antagonist, pegvisomant, did not observe regression of proliferative diabetic retinopathy. However, it has been suggested that this may have been due to insufficient suppression of IGF1.^99,100^

In diabetic nephropathy, excess GH stimulates glomerular growth, affects the structure and function of the kidney, and is associated with glomerular podocyte dysfunction.^89,101–103^

### Longevity

The GH/IGF1 axis has an intriguing association with longevity in animals. Numerous studies, particularly in rodents, indicate that suppression of the GH/IGF1 axis has multiple benefits in terms of aging. Disruption of GH and IGF1 signaling extends lifespan, enhances insulin sensitivity, decreases DNA and protein oxidation in the liver, reduces cancer incidence and may reduce age-related inflammation.^20,104–108^ GHR dysfunction clearly protects individuals with Laron Syndrome against aging-related diseases such as cancer and diabetes, but it is unclear whether GH/IGF1 deficiency increases human lifespan, although a handful of studies have indicated that attenuated GH/IGF1 function is associated with human longevity.^109,110^

## Strategies that target GHR signaling

**Fig. 4:**
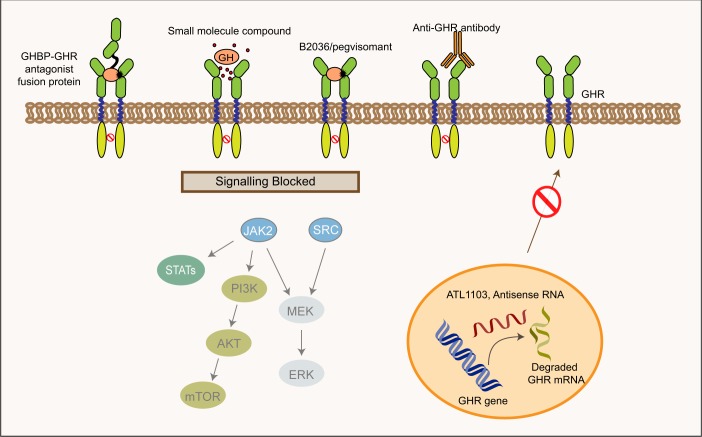
Strategies targeting the GHR One protein-derived GHR antagonist is clinically approved (pegvisomant), and several other GHR-targeted approaches are in development. These include an antagonist-GHBP fusion protein and anti-GHR antibodies, which inhibit the activation of GHR and block downstream signaling. Another approach is atesidorsen (ATL1103), an antisense oligonucleotide (ASO), that binds and induces the degradation of *GHR* mRNA. Small molecule compounds may also have applications; however, there are currently limited reports in this area

The most successful strategy to date for directly inhibiting GHR function has undoubtedly been peptide receptor antagonists, exemplified by the clinically used GHR antagonist pegvisomant (see below). However, therapeutic options to target the GHR are still limited, and pegvisomant can be difficult for researchers to access. Given the growing body of evidence that has suggested a role for this receptor in cancer and other diseases, there is certainly room for the development of alternative targeted therapeutics and several approaches are in preclinical and clinical development (Figs. 1 and 4, Table 1).

Inhibitory strategies broadly fall into three categories: those that inhibit inhibition of GH secretion from the pituitary (pre-receptor), those that directly inhibit the GHR, and drugs which inhibit downstream components of GHR signaling pathways (post-receptor).

### Inhibitors of GH secretion (pre-receptor)

Inhibitors of GH secretion include SRLs, dopamine agonizts and GHRH antagonists.^42,43,111^ SRLs bind to the somatostatin receptors that are present in the tumor and suppress the secretion of GH from the pituitary. These inhibitors include the first-generation SRLs, octreotide and lanreotide, and the second-generation SRLs, pasireotide, octreolin, and somatoprim (Table 1). Currently, SRLs are used to treat acromegaly and neuroendocrine tumors. Other applications include diabetes, obesity and cancer.^112^ SRLs also have direct anticancer activity through their actions on tumor cells that express somatostatin receptors, but it is unclear whether they suppress tumoral (autocrine/paracrine) secretion of GH.

Peptide GHRH analog antagonists have been developed in several labs. In particular, studies by Schally et al.^111^ have led to a series of well-characterized inhibitors.^113^ GHRH and GHRH receptors are expressed in many cancer cells and tumor tissues. Antagonists for GHRH inhibit the proliferation of a wide range of cancer cell lines in vitro and inhibit xenograft tumor growth, which demonstrates their potential clinical utility.^111^ Similar to SRLs, GHRH antagonists inhibit endocrine secretion of GH from the anterior pituitary to varying extents. However, the main action of these antagonists is through direct inhibition of GHRH receptors in tumor tissues. Potential applications besides oncology include acromegaly, diabetic retinopathy, and nephropathy.^111^

### GHR antagonists

#### Pegvisomant

Pegvisomant is a chemically modified (PEGylated) analog of GH that was discovered in John Kopchick’s lab and developed by Pfizer. It is the only clinically approved GHR antagonist and has been approved by the FDA for the treatment of acromegaly [reviewed in ref. ^6,114,115^]. The first reported GHR antagonist was a bovine GH protein that was mutated with three amino acid substitutions located in helix 3, which resulted in a dwarf phenotype in transgenic mice.^6,116,117^ A similar result was observed with a single amino acid substitution of glycine to arginine at position 120 on helix 3 of human GH (G120R).^118^ Pegvisomant contains a G120K mutation (substitution to a lysine also allows PEGylation at this site). Eight more mutations (H18D, H21N, R167N, K168A, D171S, K172R, E174S, and I179T) were introduced at binding site 1 on the molecule to prevent PEGylation and to increase the affinity for the receptor at this site.^6^ This antagonist, which is the protein component of pegvisomant, is known as B2036. The half-life of native GH (as well as B2036) in the circulation is short, which limits its in vivo efficacy. To reduce clearance and increase the serum half-life, polyethylene glycol-5000 (PEG5000) was conjugated to the molecule.^119^ PEGylation extends the serum half-life, delays renal clearance, and reduces the immunogenicity of pegvisomant (B2036-PEG). B2036 competes with GH in vitro on the basis of an increased affinity for the GHR. However, PEGylation reduces the affinity for the receptor somewhat. Because pegvisomant targets the GHR instead of GH, it results in reduced IGF1 and enhanced GH levels through the negative-feedback loop. Therefore, measurement of GH is not useful to monitor treatment of acromegaly with pegvisomant: instead IGF1 is determined as a surrogate biomarker.^120,121^ In addition, pegvisomant treatment decreases the insulin and glucose concentrations.^122^

Pegvisomant is highly efficacious with only mild-side effects. One concern was potential adenoma growth caused by the increased levels of circulating GH. From the clinical trials conducted so far, changes in the tumor size or recurrence were infrequent, but further assessment needs to be carried out. An evaluation of pegvisomant as long-term monotherapy in acromegalic patients from the global safety surveillance study ACROSTUDY reported increases or increases/decreases in the tumor sizes in 12 of 542 subjects (2.2%).^7^ Another study reviewed the efficacy of pegvisomant as a monotherapy for acromegaly over a 10-year period, and showed 6 of 64 (9.4%) cases with tumor growth.^123^ Pegvisomant has also been evaluated in a combination therapy with SRL, particularly with respect to normalizing the IGF1 concentration in acromegalic patients who have failed SRL monotherapy. The outcome of a 42-week study of active acromegalic patients demonstrated that the combined therapy was effective in normalizing the levels of IGF1 and that there was no indication of tumor growth.^124^ However, an analysis of 62 SRL-resistant acromegalic patients indicated better IGF1 normalization with pegvisomant monotherapy compared to combined pegvisomant/SRL treatment.^125^ Although pegvisomant is well tolerated and highly effective, some limitations need to be considered. Pegvisomant is more costly than SRL, and daily injection is required. Treatment side-effects include elevated aminotransferase levels and injection site reactions (lipohypertrophy).

As described above, a small number of preclinical studies have demonstrated efficacy for pegvisomant in certain tumor models.^71–73,76^ One obstacle to preclinical use in animal models is the species specificity of the drug. Both human GH and the protein component of pegvisomant (B2036) can bind the human and mouse GHR, but pegylation significantly reduces the affinity for the mouse GHR. Consequently, much higher concentrations of pegvisomant are necessary to reduce serum IGF1 in mice.^47^ In addition, rodent GH does not activate the human or primate GHR, so the use of pegvisomant in animal models of disease does not address the effect of the blockade of systemic GH. Therefore, although early animal studies have been promising, these limitations combine to reduce the potential of these in vivo studies to support clinical translation.

#### Antagonist–GHBP fusion proteins

An alternative approach that is used to generate long-acting forms of protein therapeutics involves generating larger chimeric proteins that avoid kidney filtration. Richard Ross and colleagues from Asterion Ltd. have developed fusion proteins composed of the GH ligand or a GHR antagonist fused to GH-binding protein (GHBP), the extracellular domain of the GHR, which is proteolytically cleaved from the receptor and exists in the circulation (Fig. 4). The fusion of GH to its natural binding protein decreases its immunogenicity and prolongs its half-life in the circulation.^126^ Initially, a GH–GHBP fusion protein agonist was generated for the treatment of GH deficiency.^127^ This chimeric protein was found to exist in solution as both a monomer and a dimer. In the dimer form, the GH portion of one molecule bound to the receptor portion of another molecule in a head-to-tail reciprocal dimer. More recently Wilkinson et al. demonstrated that fusion of GHBP to a GHR antagonist protein similar to B2036 significantly reduced IGF1 by 14% after a single subcutaneous injection in rabbits and may be useful for treating acromegaly.^128^ Introduction of a W104A mutation in the fused GHBP prevented intra- and inter-molecular binding. Three chimeric GHR antagonists were generated with extended in vivo clearance times; the terminal half-life of the fusion proteins was greater than 20 h in rats.^128^

#### Antisense oligonucleotides

Advances in antisense therapy have led to development of novel GHR antagonists (Fig. 4). Antisense oligonucleotides (ASOs) are short single-stranded DNA or RNA molecules (or chemical analogs) that bind and induce the degradation of target RNAs.^129^ Early studies reported an inhibitory effect of GHR-targeted antisense oligonucleotides on GHR expression and IGF1 production in mice.^130,131^ Antisense Therapeutics Ltd. is developing an antisense oligonucleotide drug, ATL1103 (now atesidorsen). Atesidorsen is a 20-mer ASO that has been modified to enhance its stability and circulating half-life. In Phase II outcomes, twice-weekly treatment with 200 mg atesidorsen was well tolerated and decreased the serum IGF1 concentration by 27.8% at week 14 and 18.7% at week 21 in acromegalic patients.^132^ In addition, interim analysis from a small higher dose study using twice-weekly 300 mg atesidorsen for 13 weeks demonstrated results consistent with the Phase II trial outcomes (Antisense Therapeutic Ltd.).

#### Anti-GHR antibodies

GHR antibodies that inhibit GHR-mediated signal transduction have been reported.^133–136^ Stuart Frank’s lab has developed an inhibitory conformation-sensitive monoclonal antibody (Anti-GHRcyt-mAb) that targets the extracellular domain of the rabbit GHR. Anti-GHRcyt-mAb effectively inhibits activation and downstream signal transduction of the rabbit and human GHR.^133,137^ Similarly, Sun et al.^136^ have reported a monoclonal antibody (GF185) that targets the human GHR, which acts as a full competitor for GH binding and also inhibits GHR signaling. Lan et al.^134^ have generated an anti-idiotypic antibody (CG-86) which mimics an epitope on porcine GH and investigated the inhibitory effects on GH signaling and cell proliferation, which demonstrated the potential utility of this approach for generating GH antagonists. More recently Pfizer has reported development of a humanized GHR monoclonal antagonist antibody (RN172), which blocked GH signaling in vitro and reduced IGF1 production in monkeys following a single-intravenous injection.^135^

#### Small molecules

The only small molecule GHR antagonist that has been reported is an orally available compound BVT-A (N-[5-(aminosulfonyl)-2-methylphenyl]-5-bromo-2-furamide). In two studies, BVT-A suppressed GH induction of IGF1 expression in hepatocytes in vitro and reduced GH stimulation of IGF1 secretion and body weight in hypophysectomized rats.^138,139^ However, it is unclear where in the GH signaling pathway this compound acts, and no subsequent activity has been reported in this area since the original publication.

### Inhibitors of GH signal transduction pathways (post-receptor)

Given that GH activates JAK-STAT, PI3K/AKT/mTOR and MAPK signaling, agents which target components of these signaling pathways would be expected to modulate pathway-specific GH effects. However, targeting GH signal transduction pathways will unlikely be specific to GH signaling because other receptors and cell signaling pathways can also utilize the same signaling pathways as GH. The potential for small molecules to inhibit these pathways and non-selectively inhibit GHR signaling needs to be considered in small molecule discovery studies. A detailed description of therapeutic drugs that target molecules in the GHR signal transduction pathways is beyond the scope of the current review, and we refer the reader to recent reviews.^140–144^

## Targeting autocrine/paracrine, and systemic functions: therapeutic considerations

GHR antagonism suppresses the endocrine, autocrine, and paracrine functions of GH, and this is an important consideration that has particular relevance for cancer on the basis that both systemic and tumor-derived GH contribute to cancer progression.^4,47,50^ Furthermore, it is quite clear from earlier studies by Lobie and colleagues that GH has differential effects depending on whether the source of GH is pulsatile secretion from the pituitary or secretion from cancer cells or other cells in the tumor microenvironment. For example, in breast cancer, autocrine expression of GH from the tumor cells promotes a more aggressive cellular phenotype, compared to exogenously added GH, which mimics endocrine secretion.^52,54,145,146^ This phenomenon may not be limited to cancer. For example, autocrine/paracrine actions may also be important in conditions such as diabetic retinopathy because GH expression occurs in ocular tissues.^88^ Notably, antagonism of GH signaling has the added benefit of suppressing the IGF1-mediated effects, which contribute to the etiology of several disease indications in a manner similar to that described above.

As with any therapeutic agents, the potential for side effects associated with suppression of GH should be considered. Apart from the known side effects associated with pegvisomant, which are described above, potential adverse effects may be extrapolated from adult GH deficiency (AGHD). The clinical features of AGHD includes abnormal body composition, decreased cardiac capacity, increased risk of fracture, insulin resistance, and decreased quality of life.^147–150^ The abnormal body composition is characterized by increased adipose tissue mass, decreased lean body mass, decreased muscle mass and strength.^148,149^ Cardiac changes, such as reduced aortic area and left ventricular mass index, are also reported in adult-onset GH deficiency.^151^ A study of adults with isolated GH deficiency (i.e., AGHD that does not involve abnormalities in other pituitary hormones) reported decreased total IgG levels but no increased risk of infectious disease in the subjects.^152^ In addition, abnormal vascular and neural retinal morphology was observed in adults with isolated GH deficiency.^153^ It was demonstrated that anaerobic capacity was impaired in GH-deficient adults, and this decreases exercise tolerance and quality of life.^154^ Skin and sleep changes were also reported in GH-deficient adults.^148,155^

## Conclusion

Suppression of the GH/IGF1 axis is a key medical therapy that is indicated for acromegaly and may also be useful in other diseases such as cancer. Currently, pegvisomant is the only clinically used GHR inhibitor. However, a small number of GHR antagonists are in clinical trials or preclinical development, and we anticipate that these will eventually expand the options available for clinical and research applications.

Clearly, the most successful strategy for targeting GHR signaling to date has involved protein-based therapeutics. However, other strategies are showing promise, particularly antisense oligo and antibody-based approaches. One avenue that has been underexplored is the development of small molecule therapeutic agents to target the receptor. The GHR is a challenging target in this regard in that it lacks a kinase domain and the ligand binding surface involves a large, relatively featureless protein-protein interface. Identification of inhibitors of protein-protein interactions has been a major challenge in drug discovery, yet screening strategies, including high-throughput screening and fragment-based drug design, are able to identify compounds suitable for drug development.^156,157^ Recent successes with such strategies have resulted in high impact therapies including agents that block the p53-MDM protein interaction and the formation of antiapoptotic complexes by the Bcl2 protein with Navitoclax and Venetoclax.^157^ Conceivable mechanisms for on-target GHR inhibition involve disruption of the formation of the signaling complex. Indeed, this type of inhibitory mechanism was described for blocking the interaction between another cytokine receptor, the interleukin-2 receptor, and its ligand.^158^ Recent advances in our understanding of GHR activation^24^ should contribute to the development of small molecule-based targeting strategies.
